# Association of Insulin Resistance, Arterial Stiffness and Telomere Length in Adults Free of Cardiovascular Diseases

**DOI:** 10.1371/journal.pone.0136676

**Published:** 2015-08-26

**Authors:** Irina Strazhesko, Olga Tkacheva, Sergey Boytsov, Dariga Akasheva, Ekaterina Dudinskaya, Vladimir Vygodin, Dmitry Skvortsov, Peter Nilsson

**Affiliations:** 1 Department of aging and age-associated diseases prevention, National Research Center for Preventive Medicine, Moscow, Russian Federation; 2 Department of Clinical Cardiology and Molecular Genetics, National Research Center for Preventive Medicine, Moscow, Russian Federation; 3 Department of Epidemiology of Chronic Non-Communicable Diseases Laboratory of Biostatistics, National Research Center for Preventive Medicine, Moscow, Russian Federation; 4 Department of Chemistry, Lomonosov Moscow State University, Moscow, Russian Federation; 5 Department of Clinical Sciences, Lund University, Skane University Hospital, Malmo, Sweden; University of Catania, ITALY

## Abstract

**Background:**

Chronic inflammation and oxidative stress might be considered the key mechanisms of aging. Insulin resistance (IR) is a phenomenon related to inflammatory and oxidative stress. We tested the hypothesis that IR may be associated with cellular senescence, as measured by leukocyte telomere length (LTL), and arterial stiffness (core feature of arterial aging), as measured by carotid-femoral pulse wave velocity (c-f PWV).

**Methods:**

The study group included 303 subjects, mean age 51.8 ±13.3 years, free of known cardiovascular diseases and regular drug consumption. For each patient, blood pressure was measured, blood samples were available for biochemical parameters, and LTL was analyzed by real time q PCR. C-f PWV was measured with the help of SphygmoCor. SAS 9.1 was used for statistical analysis.

**Results:**

Through multiple linear regression analysis, c-f PWV is independently and positively associated with age (p = 0.0001) and the homeostasis model assessment of insulin resistance (HOMA-IR; p = 0.0001) and independently negatively associated with LTL (p = 0.0378). HOMA-IR seems to have a stronger influence than SBP on arterial stiffness. In all subjects, age, HOMA-IR, LTL, and SBP predicted 32% of the variance in c-f PWV. LTL was inversely associated with HOMA-IR (p = 0.0001) and age (p = 0.0001). In all subjects, HOMA-IR, age, sex, and SBP predicted 16% of the variance in LTL.

**Conclusions:**

These data suggest that IR is associated with cell senescence and arterial aging and could, therefore, become the main target in preventing accelerated arterial aging, besides blood pressure control. Research in telomere biology may reveal new ways of estimating cardiovascular aging and risk.

## Introduction

Aging is the primary marker influencing risk of cardiovascular disease (CVD), which is largely the result of dysfunctional arteries and superimposed atherosclerosis. In 2008 the concept of early vascular ageing (EVA) was introduced [1]. The core feature of EVA is arterial stiffness, measured as increased carotid-femoral pulse wave velocity (c-f PWV) in relation to a subject’s chronological age and sex [2]. c-f PWV has been shown to be an independent risk marker for both cardiovascular events and overall mortality in hypertensive patients [3] and the elderly [4], as well as in glucose tolerance-tested study groups [5]. A number of factors are responsible for increasing arterial stiffness, namely: decreased elastin and increased collagen in the arterial wall, abnormal endothelial regulation of arterial smooth muscle tone, and the accumulation of advanced glycosylation end products (AGEs) leading to protein cross-linking [6]. Results of cross-sectional studies have associated aortic stiffness in particular with obesity, impaired glucose tolerance, type 2 diabetes mellitus (T2DM) [7], different clusters of metabolic syndrome [8]. It is possible that the majority of the additional risk of CVD in T2DM is mediated through pathophysiological mechanisms involving increased arterial stiffness [9]. In addition to the effects of AGEs, insulin and/or insulin resistance (IR) may contribute to the development of arterial stiffness [10]. IR has been associated with arterial stiffness, independent of glucose tolerance status [11].

It has been shown that variables other than hormones or metabolic indicators e.g. leukocyte telomere length (LTL), the marker of replicative cellular senescence, might determine or reflect the vessels’ biological age [12]. Telomeres are the TTAGGG tandem repeats at the ends of chromosomes protecting the chromosomal ends from erosion during cell division. Telomerase plays the key role in maintaining the telomere length. Individuals with short telomeres are more likely to show accelerated vascular aging [13,14], atherosclerosis [15], coronary heart disease [16] and T2DM [17]. But there is still uncertainty about the role of telomere biology in increased arterial stiffness [18] and the existence of common pathophysiological mechanisms involving arterial aging and replicative cellular senescence.

The loss of LTL is accelerated by chronic inflammation and oxidative stress [19]. LTL reflects both an individual’s telomere length at birth and the telomere attrition during the life course, demonstrating replicative history and cumulative oxidative burden [20]. Little information is available on the relationship of LTL or telomeres attrition rates in relation to cardiovascular risk factors such as impaired fasting glucose (IFG) and IR–a phenomenon probably related to inflammatory and oxidative stress status. A recent study in young adults showed individual telomeres attrition rates to be highly variable and strongly correlated with IR [21].

We tested the hypothesis that IR is independently associated with LTL and c-f PWV. An additional aim of our study was to determine whether LTL, a possible index of biological aging, explains some of the variability in aortic stiffness.

## Materials and Methods

### Study Design

By advertisement we recruited 450 subjects who visited the National Research Center for Preventive Medicine in Moscow, Russia, from May 2012 to December 2012. To determine eligibility, subjects completed a health screening which included their medical history, a physical examination, and a blood sampling for laboratory analyses. We excluded 147 subjects with previous history of drug medication for diabetes, hypertension or hyperlipidemia; a history of stroke, coronary heart disease, peripheral arterial disease, arrhythmia, congestive heart failure, or valvular heart disease; hepatic or kidney failure, as well as a cancer. Following these exclusions, 303 subjects were included in the study.

The study was approved by the Independent Ethics Committee of the National Research Center For Preventive Medicine, Moscow, Russia. Informed written consent was obtained from all subjects prior to inclusion in the study.

Systolic blood pressure (SBP) and diastolic blood pressure (DBP) were measured after resting for more than five minutes according to a standardized operating procedure using a calibrated sphygmomanometer and brachial inflation cuff (HEM-7200 M3, Omron Healthcare, Kyoto, Japan). The mean of three consequent readings was accepted.

Anthropometric measurements were used to calculate the body mass index (BMI, kg/m^2^). After an overnight fast, serum fasting glucose (FG) and glycosylated hemoglobin (HbA_1c_) were determined using routine laboratory methods on a biochemical analyzer "Sapphire 400" (Niigata Mechatronics, Japan).

Serum insulin was quantified using the chemiluminescent microparticle on the Immunoassay analyzer “Architect i 2000SR» (Abbot, Canada) [22]. Homeostasis model assessment of insulin resistance (HOMA-IR) = fasting insulin (mU/ml) x FG (mmol/l)/22.5.

Impaired fasting glucose (IFG) was diagnosed if FG ≥ 6.1 and <7.0mmol/l. During an oral glucose tolerance test (OGTT) fasting glucose was measured at baseline and post/challenge glucose after administration of 75 g of glucose at 120 min. Subjects were categorized into normal glucose tolerance (2-h glucose level <7.8 mmol/l) and impaired glucose tolerance (IGT): 2-h glucose level 7.8–11,0 mmol/l following the screening OGTT.

### Arterial stiffness

Arterial stiffness was assessed according to the c-f PWV values. It was measured using the SphygmoCor 8.0 hardware (Atcor, Sydney) with the help of an applanation tonometer and electrocardiogram gating to attain pulse waves from both proximal (carotid artery) and distal (femoral artery) sites. The c-f PWV was calculated from the transit time between the two sites relative to the R-wave within the electrocardiogram complex using the ‘foot-to-foot method’ and the intersecting tangent algorithm [23]. In each subject two sequences of measurements were performed, and their mean value was considered for analysis. The repeatability coefficient value was 0.935.

### Leukocyte telomere length analysis

LTL was determined according to the method described by Cawthon [24]. Genomic deoxyribonucleic acid (DNA) was extracted directly from blood samples by standard procedures (OD_260nm/280nm_ 1.8–1.9). The assay involved comparing the abundance of telomere DNA to the single copy genomic DNA number for each sample and by further comparison of normalized value between DNAs of different sources. Ratio of the telomere (T) and single-copy *36B4* gene(S) matrices reflect the length of telomeres (the T/S ratio is approximately [2^C^_t_ (telomeres)/2^*C*^_t_(^36B4)^]^-1^ = 2^-ΔC^_t_ [T1]). Simultaneously stock mix 1,25 x (1x mixture: PCR buffer 1x (Fermentas 10X PCR Hotstartbuf + KCl), MgCl2 2 mM, dNTP 0.2 mM, 0.5 μM of each primer, 0.05 units / μl of Taq polymerase Maxima (Fermentas), Sybr Green I 0.2x) have been prepared. The primer sequences were: Tel1, GGTTTTTGAGGGTGAGGGTGAGGGTGAGGGTGAGGGT, Tel2, TCCCGACTATCCCTATCCCTATCCCTATCCCTATCCCTA., 36B4u, CAAGTGGGAAGGTGTAATCC, 36B4d, CCCATTCTATCATCAACGGGTACAA. Sixteen microliters of master mix were added to each sample well and 4 μl of the analyzed genomic DNA with a concentration of 10 ng/μl was added. Samples were mixed, centrifuged, and amplified in a thermocycler CFX96. For telomere polymerase chain reaction (PCR), we then heated at 95C for 5 minutes, and did 35 cycles of 95C for 20 sec, 54C for 2 minutes, followed by melting. For control PCR we heated at 95 C for 5 minutes. Then we did 35 cycles of 95C for 20 sec, 58C for 1 minute, followed by melting. The amplification of the corresponding telomeric and control mixtures occupy one cell unit. For each sample, we did three repetitive telomeric reactions and three control reactions. We calculated the difference between cycle thresholds of amplification of the telomere and single copy of the gene (ΔC_t_), and based on these results appreciated relative telomere lengths. The genomic DNA of the cell HEK line and control leukocyte sample was used as a reference point. To take into account differences in PCR mixtures from time to time the we set the leukocyte reference ΔC_t(leu)_ value at 8. Relative exponential length L was set L = ΔC_t_-(ΔC_t(leu)_-8). As we do not get the absolute value of the lengths of telomeres, so as a measure of the spread of values, it was decided to use the standard deviation. In our experiment, the standard deviation in almost all cases was in the range of 0.1–0.4 derived from the relative lengths of 8.30 to 11.39 (logarithmic scale).

### Telomerase activity analysis

Telomerase activity (TA) was measured using the method described by Kim [25]. The analysis of cellular extract from monocyte fraction of white blood cells (erythrocytes prevents impurity analysis) containing 2 mcg of total protein was performed. The cells, derived from the monocytic ring on Ficoll density gradient and washed with PBS were re-suspended in lytic buffer (10 mMTris-HCl and 10 mM HEPES-KOH, pH 7.5, 1.0 mM MgCl2, 1 mM EGTA, 5 mM β-mercaptoethanol, 5% glycerol, 0.5% CHAPS, 0.1 mM PMSF). The cells were incubated for 30 minutes on ice, centrifuged for 10 minutes at 4°C for 15 000 g, and the supernatant solution was collected. The extract was aliquoted and frozen in liquid nitrogen. The telomerase polymerase reaction was carried out with *24*μl of 1,2x master mix (1x mix contains *1X TRAP-buffer (1X TRAP-buffer*: *20 mM HEPES-KOH pH 8*.*3*, *1*,*5*mM MgCl2, 63 mMKCl, 1mM EGTA, 0,1 mg / ml BSA, 0,005% v / v Tween-20), 20 pM of dNTP, 10 pmol of oligonucleotide TS (AATCCGTCGAGCAGAGTT) and 4 μl monocyte or control extract. The reaction mixture was incubated for 30 minutes at 25°C. The products were amplified by PCR in real time. Thereafter 1.5 units of Taq-DNA polymerase ("Helicon"), 10 pmol of oligonucleotide ACX (CGCGGCTTACCCTTACCCTTACCCTTACC) and Sybr Green I to 0.2x final concentration in the mixture were added in ice (together 2 μl volume). Real time PCR was carried out on the device CFX-96 for 35 s at 94°C, 35 s at 50°C, 90 s at 72°C (30 cycles of thermal cycler Mastercycler ("Bio-Rad")). As a calibration curve a series of dilutions of cell extracts of HEK cell line (15 cells activity was set as 1) and TSR8 (sequence identical to the TS primer extended with 8 telomeric repeats AG(GGTTAG)_7_) has been used.

### Statistical Analysis

SAS 9.1 was used for statistical analysis (SAS Institute, Cary, NC, USA). Mean values ± standard deviations (SD) for continuous clinical characteristics and the corresponding proportions/frequencies for categorical data were computed and presented in tables. The distributions were compared by using one-way ANOVA for continuous variables, as well as the Chi-square test and t-test (with Fisher's arcsine-transformation) for categorical/binary variables. The Pearson’s linear correlations and Spearman’s rank correlation coefficients were calculated to evaluate the bivariate relationships—between c-f PWV and LTL, as well as clinical variables. A multiple linear regression analysis was performed to identify any independent associations between c-f PWV and parameters of glucose metabolism plus LTL and between LTL and parameters of glucose metabolism. P values less than 0.05 were considered statistically significant.

## Results

### Characteristics of study subjects

A total of 303 ambulatory participants (104 males and 199 females) were recruited. Subjects did not differ in overall ethnic composition identifying as Caucasians. TA data were available for 163 subjects, OGTT was made in 231 subjects, and HOMA-IR data were calculated for 274 subjects. Other data were available for all participants. The subjects ranged between 23 and 91 years of age, with a mean age of 51.8 ±13.3 years. Of the study group, 76 subjects had mild or moderate hypertension, 50 subjects had T2DM, and 33 subjects were diagnosed with IGT. None of the patients with T2DM regularly received anti-diabetes medication, and none had known microvascular or macrovascular complications. None of the patients regularly received any other medication including antihypertensive drugs.

The study sample was divided into two groups according to the HOMA-IR level. IR was diagnosed in case of HOMA-IR elevation > 2.5 [26]. According to these criteria IR was diagnosed in 89 subjects. Table 1 represents the parameters of interest in total group and in the subgroups categorized by IR status. Data are presented with Mean values ± standard deviations (SD).

**Table 1:** Clinical and metabolic characteristics of the study participants in the total group and according to HOMA-IR.

	Total group n = 303	HOMA-IR ≤ 2.5 n = 185	HOMA-IR >2.5 n = 89	P-value
Age (years)	51.5±13.3	50.3±13.4	52.7±11.6	0.136
Men, n (%)	104 (34.3%)	56 (30.3%)	41 (46.1%)	0.012
BMI (kg/m^2^)	27.4±5.2	25.7±4.3	31.5±4.8	< 0.0001
SBP (mmHg)	125±16	122±17	133±13	< 0.0001
DBP (mmHg)	78±10	77±10	83±10	< 0.0001
c-f PWV (m/s)	11.96±2.61	10.46±2.34	12.01±2.93	< 0.0001
FG (mmol/l)	5.8±1.4	5.3±0.8	6.8±1.9	< 0.0001
2hOGTT(mmol/l)	5.7±1.8	5.4±1.6	6.6±2.2	0.0004
HbA_1c_ (mmol/l)	5.5±1.0	5.3±0.7	6.1±1.2	< 0.0001
LTL	9.76±0.50	9.86±0.46	9.61±0.47	< 0.0001
TA	0.59±0.39	0.63±0.38	0.55±0.42	0.249

**Abbreviations:** BMI: body mass index; c-f PWV: carotid-femoral pulse wave velocity; DBP: diastolic blood pressure; FG: fasting glucose; HbA_1c_: glycosylated hemoglobin; HOMA-IR: homeostasis model assessment of insulin resistance; LTL: leukocyte telomere length; SBP: systolic blood pressure; TA: telomerase activity; 2h OGTT:2-h glucose level following the oral glucose tolerance test; P-value: p between HOMA-IR ≤ 2.5 and HOMA-IR >2.5 groups

Compared to subjects with normal HOMA-IR, those with elevated HOMA-IR had a higher BMI (p <0.0001), FG (p < 0.0001), 2h-OGTT glucose (p = 0.0004), HbA_1c_ (p <0.0001), SBP (p <0.0001), DBP (p <0.0001), c-f PWV (p <0.0001), and shorter LTL (p <0.0001). There was higher proportion of men in the “high” HOMA-IR group.

Subjects with IR (elevated HOMA-IR) did not significantly differ from those with normal HOMA-IR in age and TA.

### Bivariate correlations

Table 2 represents the summary of c-f PWV and LTL bivariate associations. Pearson’s correlation coefficients were used and the results were verified using more robust Spearman’s rank correlation coefficients.

**Table 2:** Bivariate associations between c-f PWV and LTL in relation to age, BP and glucose metabolism.

Variable		c-f PWV(m/s)		LTL	
		r	p	r	p
Age (years)	PLC	0.456	0.0001	-0.295	0.0001
	SRC	0.448	0.0001	-0.319	0.0001
Sex	PLC	0.036	0.543	-0.111	0.0542
	SRC	0.076	0.2040	-0.125	0.0309
SBP mmHg	PLC	0.327	0.0001	-0.119	0.0394
	SRC	0.348	0.0001	-0.145	0.0124
DBP mmHg	PLC	0.19	0.0013	-0.052	0.3753
	SRC	0.222	0.0002	-0.034	0.5623
FG (mmol/l)	PLC	0.425	0.0001	-0.249	0.0001
	SRC	0.375	0.0001	-0.212	0.0002
2h OGTT (mmol/l)	PLC	0.284	0.0001	-0.118	0.0709
	SRC	0.229	0.0005	-0.122	0.0608
HOMA-IR	PLC	0.333	0.0001	-0.31	0.0001
	SRC	0.333	0.0001	-0.271	0.0001
HbA_1c_ (mmol/l)	PLC	0.378	0.0001	-0.159	0.0064
	SRC	0.226	0.0001	-0.179	0.0022
TA	PLC	-0.158	0.0354	0.098	0.188
	SRC	-0.191	0.0109	0.166	0.0246
LTL	PLC	-0,270	0.0001		
	SRC	-0.327	0.0001		

Abbreviations: c-f PWV: carotid-femoral pulse wave velocity; DBP: diastolic blood pressure; FG: fasting glucose; HbA_1c_: glycosylated hemoglobin; HOMA-IR: homeostasis model assessment of insulin resistance; LTL: leukocyte telomere length; PLC; Pearson’s linear correlation coefficients; SBP: systolic blood pressure; SRC: Spearman’s rank correlation coefficients; TA: telomerase activity; 2h OGTT: 2-h glucose level following the oral glucose tolerance test

According to the results of Pearson’s linear correlation test arterial stiffness (c-f PWV) was significantly and positively correlated with age (p = 0.0001), SBP (p = 0.0001), DBP (p = 0.0013), FG (p = 0.0001), HOMA-IR (p = 0.0001), 2h-OGTT glucose (p = 0.0001) and HbA_1c_ (p = 0.0001). A significant inverse correlation was identified between c-f PWV and LTL (p = 0.0001), between c-f PWV and TA (p = 0.0354). No correlation was found between c-f PWV and sex.

LTL was significantly negatively correlated with age (p = 0.0001), SBP (p = 0.0394), FG (p = 0.0001), HOMA-IR (p = 0.0001), HbA_1c_ (p = 0.0064), marginally negatively correlated with male sex (p = 0.0542). It tended to be negatively correlated with 2-h OGTT glucose (p = 0.0709) but not at all with DBP (p >0.1). LTL was significantly positively correlated with TA (p = 0.0246) when Spearman’s rank correlation coefficients were used.

### Multiple regression analysis

The first step in building a multiple regression was to identify the explanatory variables (which were significantly related to the dependent variable) independently from age and sex for LTL, as well as age and SBP for c-f PWV. For this purpose we used explanatory variables that have demonstrated the high bivariate correlations with dependent variable, respectively. We included one explanatory variable in each model—at the mandatory inclusion of sex and age with their interaction for variable LTL, as well as the age and SBP with their interaction for variable c-f PWV (see S1 Table and S2 Table). All studied parameters of glucose metabolism (FG, HOMA-IR, HbA_1c_, 2-h OGTT glucose) and LTL may be considered as independent variables associated with c-f PWV, adjusted for age and SBP. Only HOMA-IR and FG may be considered as independent variables associated with LTL, adjusted for age and sex.

At the second step we used a multiple regression model to determine the independent effect of HOMA-IR on vascular stiffness (c-f PWV) with adjustment for age, LTL, SBP and the independent effect of HOMA-IR on LTL with adjustment for age, sex, SBP. To assess the normality of the outcome variables histograms of c-f PWV and LTL were constructed (S1 Fig and S2 Fig).

The first model of multiple linear regression analysis used the c-f PWV as the dependent variable and age, SBP, LTL, HOMA-IR as independent variables (Table 3).

**Table 3:** Multiple linear regression analysis of c-f PWV (dependent variable) on age, SBP, LTL, HOMA-IR as independent variables.

Predictor	β ± S.E.	Type III SS	P	Error SS	Total SS	Model R^2^
*Intercept*	*6*.*271±0*.*596*	*566*.*965*	*0*.*0001*			
Age	0.090±0.011	326.396	0.0001	1279.289	1605.685	0.2033
*Intercept*	*17*.*227±3*.*177*	*143*.*930*	*0*.*0001*			
Age	0.080±0.011	243.908	0.0001			
LTL	-1.068±0.304	60.249	0.0005	1219.041	1605.685	0.2408
*Intercept*	*14*.*099 ±3*.*323*	*85*.*739*	*0*.*0001*			
Age	0.070±0.012	166.021	0.0001			
LTL	-1.013±0.301	53.926	0.0009			
SPB	0.025±0.009	38.154	0.0050	1180.887	1605.685	0.2646
*Intercept*	*10*.*852 ±3*.*282*	*48*.*326*	*0*.*0011*			
Age	0.072±0.011	176.972	0.0001			
LTL	-0.631±0.302	19.280	0.0378			
SBP	0.013 ±0.009	10.417	0.1260			
HOMA-IR	0.327±0.073	89.158	0.0001	1091.729	1605.685	0.3201

Abbreviations: c-f PWV, carotid-femoral pulse wave velocity; Error SS: error of sum of squares; HOMA-IR, homeostasis model assessment of insulin resistance; LTL, leukocyte telomere length; SBP, systolic blood pressure; S.E.: standard error; **Total SS: total sum of squares;** Type III SS:type III sum of squares.

Age significantly accounted for 20.3% of the c-f PWV variability. A regression model with age and LTL accounted for 24.1% of the c-f PWV variability, with the additional 3.8% variation accounted for LTL. A model that evaluated age, LTL and SBP explained 26.5% of the c-f PWV variability with the additional 2.4% variation explained by SBP. When age, LTL, SBP, HOMA-IR were all introduced into the model, HOMA-IR was accounted for additional 5,5% variability in c-f PWV. This analysis indicated a marked effect of HOMA-IR on c-f PWV over and above the effect of SBP.

The second model of multiple linear regression analysis used the LTL as the dependent variable and age, sex, SBP, HOMA-IR as independent variables (see Table 4).

**Table 4:** Multiple linear regression analysis of LTL (dependent variable) with age, sex, SBP, HOMA-IR as independent variables.

Predictor	β ± S.E.	Type III SS	P	Error SS	Total SS	Model R^2^
*Intercept*	*10*.*266±0*.*116*	*1682*.*573*	*0*.*0001*			
Age	-0.009±0.002	3.955	0.0001	56.504	60.459	0.0654
*Intercept*	*10*.*39 ±0*.*122*	*1503*.*580*	*0*.*0001*			
Age	-0.011±0.002	4.855	0.0001			
Sex	-0.167±0.060	1.624	0.0056	54.880	60.459	0.0923
*Intercept*	*10*.*427±0*.*220*	*469*.*628*	*0*.*0001*			
Age	-0.01±0.002	4.025	0.0001			
Sex	-0.167±0.062	1.433	0.0093			
SBP	-0.0004±0.002	0.010	0.828	54.870	60.459	0.0924
*Intercept*	*10*.*287±0*.*214*	*447*.*997*	*0*.*0001*			
Age	-0.010±0.002	3.551	0.0001			
Sex	-0.126±0.060	0.843	0.0377			
SBP	0.002±0.002	0.161	0.3625			
HOMA-IR	-0.067±0.014	4.222	0.0001	50.649	60.459	0.1623

Abbreviations: Error SS: error of sum of squares; HOMA-IR, homeostasis model assessment of insulin resistance; LTL, leukocyte telomere length; SBP, systolic blood pressure; S.E.: standard error; **Total SS: total sum of squares;** Type III SS: type III sum of squares

A model that evaluated age, sex, SBP and HOMA-IR had explained 16.2% of the LTL variability. HOMA-IR significantly accounted for 7.0% of the LTL variability. When age, sex, HOMA-IR and SBP were all introduced into the model, SBP was not significantly associated with variability in LTL (p *>*0.3).

S3 and S4 Figs display scatter plots of c-f PWV and LTL as a function of HOMA-IR.

## Discussion

The important finding of our observational study is that markers of glucose metabolism (FG, HbA_1c_, 2h OGTT glucose level, HOMA-IR) are all associated with arterial stiffness. Most of our patients do not have T2DM (and none drug treated) and the values of glucose metabolism parameters are in the normal range. Our results are in accordance with findings that FG level, even within the normal range, is associated with aggravation of arterial stiffness in non-diabetic healthy subjects [27]. Lukich *et al*. reported that 284 Caucasian subjects showed a positive correlation among FG, HbA_1c_ and PWV, and that increased arterial stiffness started at the IFG level according to comparisons of normal glucose, IFG, and diabetes [28]. These results support the hypothesis that high-normal FG level is associated with target organ damage and vascular dysfunction, independent of other factors including blood pressure, and explain why high normoglycemic status is a risk factor of CVD. In addition, Bjornholt JV et al. demonstrated after twenty-two years of follow- up that non-diabetic men with FG level > 85 mg/dl had a 1.4-fold higher risk of cardiovascular death than did men with lower FG [29].

Our results confirm the hypothesis that an important glucometabolic component of increased arterial stiffness occurs before the onset of T2DM. Such alterations may be caused by factors such as carbonyl and oxidative stress, chronic low-grade inflammation, and endothelial dysfunction, including that caused by long-term hyperglycemia and formation of AGEs [30].

IR seems to be one of the most important factors influencing arterial stiffness. The mechanism underlying the relationship between IR and arterial stiffness is unknown. Some other studies also found a relationship between IR and arterial stiffness in both patients with diabetes and healthy young individuals [31]. As a factor influencing arterial stiffness, the effect of insulin *per se* is of potential importance and not definitively established. Insulin has inducted vascular smooth muscle proliferation and migration in cell culture [32]. Unresponsiveness of endothelium-mediated vasodilation associated with IR could explain the link to arterial stiffness [33]. These factors may contribute to arterial stiffness before impaired glucose tolerance or diabetes has developed [10]. Further research is needed to resolve the roles played by hyperinsulinemia and/or IR in the progression of arterial stiffness, and to determine whether endothelial dysfunction or vascular smooth muscle proliferation mediate the observed association. Additional studies are also needed to assess the effect of reversibility, i.e. improved insulin sensitivity as a way to decrease arterial stiffness. It is very important to stress that HOMA-IR seems to have a stronger influence on arterial stiffness than SBP (Table 3). Our findings support the notion of Bernhard M. et al. [34] that vascular stiffness is a precursor rather than the result of hypertension.

The second main finding showed that c-f PWV was inversely correlated with LTL, as a suggested marker of biological aging. This supports that individuals who are characterized by relatively shorter telomeres manifest a relatively higher c-f PWV. The relation between LTL and c-f PWV might hold not only for telomeres in leukocytes but also for telomeres in other replicating cells, including vascular endothelial cells and vascular smooth muscle cells. Such findings link the biologic aging of major blood vessels to the aging of cellular elements of the vascular wall.

The third main finding was that LTL was inversely associated with age, HOMA-IR and was shorter in men compared with women. These results are in accordance with others [21]. Cross-sectional analyses have demonstrated that LTL in leukocytes correlates inversely with age. It is known that at birth telomeres are of the same length in boys and girls; later in life, however, they are relatively longer in women, especially in the premenopausal years compared with corresponding men, an effect attributed to estrogen [12]. However, some of our results were unexpected. The influence of HOMA-IR was comparable in value with the influence of age on LTL (Table 4). The explanation may be the following. IR is supposed to increase oxidative stress [35]. Oxidative stress and inflammation are considered important factors influencing the biology of aging. Oxidative stress causes single-strand breaks specific to telomeres and elevates nuclear removal of the telomerase reverse transcriptase. Both processes accelerate telomere erosion. Chronic inflammation, which is associated with an increased leukocytes turnover, is also linked to accelerated telomere attrition. Both LTL and TA reflect the functional state of stem progenitor cells [36]. IR linked with chronic inflammation can enhance telomere shortening in stem cells and a subsequent decrease in their functional capacity. Stem and progenitor cells are involved in repairing damaged tissue and the differentiation processes. Thus, they are important in maintaining tissue homeostasis, including that of the vessel wall. We hypothesize that LTL reflects the association between IR and arterial stiffness and may be regarded as a component linked to accelerated aging.

Finally, we acknowledge potential limitations of our study. As this study was cross-sectional we simultaneously collected data on c-f PWV, telomeres, glucose regulation, and blood pressure. Such a cross-sectional approach does not reveal cause–effect relationships. Further research is needed to understand the mechanisms that underlie these associations, for example by use of interventions, and to determine the optimal glycemic target value for the prevention of arterial stiffness in clinical and public health settings.

In conclusion, increased arterial stiffness is associated with shorter telomere length and impaired glucose metabolism. Short LTL and impaired glucose metabolism may be considered as non-hemodynamic components of EVA. IR is associated with arterial stiffness and LTL and could therefore become the main target in preventing accelerating arterial aging, besides blood pressure control. Research in telomere biology may reveal new ways of estimating cardiovascular aging and risk.

## Supporting Information

S1 Table Multiple linear regression analysis of c-f PWV (dependent variable) on FG, HbA_1c_, HOMA-IR, 2h OGTT, LTL,TA as independent variables, being adjusted by Age, SBP and the interaction term of Age*SBP. (DOCX)

S2 Table Multiple linear regression analysis of LTL (dependent variable) on HOMA-IR, FG, HbA_1c_, SBP as independent variables, being adjusted by Age, Sex and the interaction of Age*Sex. (DOCX)

S1 Fig Histogram plot of carotid-femoral pulse wave velocity (c-f PWV) values distribution. (DOCX)

S2 Fig Histogram plot of leukocyte telomere length (LTL) values distribution. (DOCX)

S3 Fig Scatter plots of carotid-femoral pulse wave velocity (c-f PWV) as a function of Homeostasis model assessment of insulin resistance (HOMA-IR). (DOCX)

S4 Fig Scatter plots of leukocyte telomere length (LTL) as a function of Homeostasis model assessment of insulin resistance (HOMA-IR) (DOCX)
