# Supplementary material for: Association of Insulin Resistance, Arterial Stiffness and Telomere Length in Adults Free of Cardiovascular Diseases
Source: PLoS One. 2015 Aug 26;10(8):e0136676. doi: 10.1371/journal.pone.0136676 (PMC4550423; doi:10.1371/journal.pone.0136676)
Supplement: S1 Table — (DOCX) [file pone.0136676.s001.docx]

**S1 Table.** Multiple linear regression analysis of c-f PWV (dependent variable) on FG, HbA_1c,_ HOMA-IR, 2h OGTT, LTL,TA as independent variables, being adjusted by Age, SBP and the interaction term of Age*SBP.

| **Predictor** | **β ± S.E.** | Type III SS | **P** | **Model R^2^** |
| --- | --- | --- | --- | --- |
| **Model 1** |  |  |  |  |
| *Intercept* | *-1.399±1.517* | *3.871* | *0.3573* |  |
| Age | 0.072±0.011 | 190.564 | 0.0001 |  |
| SBP | 0.045±0.012 | 61.743 | 0.0003 |  |
| Age*(SBP=140+) | -0.021±0.008 | 28.940 | 0.0122 |  |
| **FG** | **0.563±0.093** | **168.246** | **0.0001** | **0.3433** |
|  |  |  |  |  |
| **Model 2** |  |  |  |  |
| *Intercept* | *-0.0519±1.451* | *0.004* | *0.9722* |  |
| Age | 0.071±0.010 | 159.723 | 0.0001 |  |
| SBP | 0.050±0.012 | 62.731 | 0.0001 |  |
| Age*(SBP=140+) | -0.023±0.009 | 24.37 | 0.0089 |  |
| **2h OGTT** | **0.203±0.071** | **28.407** | **0.0048** | **0.3216** |
|  |  |  |  |  |
| **Model 3** |  |  |  |  |
|  |  |  |  |  |
| *Intercept* | *-0.788±1.611* | *1.153* | *0.6252* |  |
| Age | 0.086±0.011 | 262.689 | 0.0001 |  |
| SBP | 0.042±0.013 | 46.926 | 0.0020 |  |
| Age*(SBP=140+) | -0.022±0.009 | 28.509 | 0.0157 |  |
| **HOMA-IR** | **0.312 ±0.063** | **117.749** | **0.0001** | **0.3190** |
|  |  |  |  |  |
| **Model 4** |  |  |  |  |
|  |  |  |  |  |
| *Intercept* | *-2.121±1.620* | *8.182* | *0.1918* |  |
| Age | 0.068±0.112 | 164.571 | 0.0001 |  |
| SBP | 0.051±0.012 | 76.127 | 0.0001 |  |
| Age*(SBP=140+) | -0.022±0.009 | 30.356 | 0.0123 |  |
| **HbA_1c_** | **0.643 ±0.141** | **99.111** | **0.0001** | **0.3051** |
|  |  |  |  |  |
| **Model 5** |  |  |  |  |
| *Intercept* | *8.721 ±3.516* | *30.925* | *0.0001* |  |
| Age | 0.076±0.011 | 206.896 | 0.0001 |  |
| SBP | 0.048±0.013 | 66.583 | 0.0003 |  |
| Age*(SBP=140+) | -0.018±0.008 | 19.339 | 0.0508 |  |
| **LTL** | **-0.763 ±0.292** | **34.189** | **0.0096** | **0.2699** |
|  |  |  |  |  |
| **Model 6** |  |  |  |  |
| *Intercept* | *2.227±2.084* | *5.73* | *0.2867* |  |
| Age | 0.095±0.0151 | 209.340 | 0.0001 |  |
| SBP | 0.037±0.017 | 24.684 | 0.0280 |  |
| Age*(SBP=140+) | -0.021±0.011 | 18.094 | 0.0595 |  |
| **TA** | **-0.582±0.446** | **8.540** | **0.1943** | **0.2617** |

Abbreviations: c-f PWV: carotid-femoral pulse wave velocity; FG: fasting glucose; HbA_1c_: glycosylated hemoglobin; HOMA-IR: homeostasis model assessment of insulin resistance; LTL: leukocyte telomere length; SBP: systolic blood pressure; S.E.: standard error; Type III SS: type III sum of squares; TA: telomerase activity; 2h OGTT: 2-h glucose level following the oral glucose tolerance test.

The interaction between age and SBP was calculated by multiplication of age by the binary variable depending on the fact of SBP increase over 140 mm Hg.
