# Supplementary material for: Association of Insulin Resistance, Arterial Stiffness and Telomere Length in Adults Free of Cardiovascular Diseases
Source: PLoS One. 2015 Aug 26;10(8):e0136676. doi: 10.1371/journal.pone.0136676 (PMC4550423; doi:10.1371/journal.pone.0136676)
Supplement: S2 Table — (DOCX) [file pone.0136676.s002.docx]

**S2 Table.** Multiple linear regression analysis of LTL (dependent variable) on HOMA-IR, FG, HbA_1c,_ SBP as independent variables, being adjusted by Age, Sex and the interaction of Age*Sex.

| **Predictor** | **β ± S.E.** | Type III SS | **P** | **Model R^2^** |
| --- | --- | --- | --- | --- |
| **Model 1** |  |  |  |  |
| *Intercept* | *10.458±0.143* | *1043.502* | *0.0001* |  |
| Age | -0.009±0.003 | 2.573 | 0.0003 |  |
| Sex | -0.162±0.232 | 0.095 | 0.4848 |  |
| Age*Sex | -0.001±0.005 | 0.009 | 0.08286 |  |
| **HOMA-IR** | **-0.057±0.013** | **4.040** | **0.0001** | **0.1603** |
|  |  |  |  |  |
| **Model 2** |  |  |  |  |
| *Intercept* | *10.749±0.160* | *958.565* | *0.0001* |  |
| Age | -0.012±0.002 | 4.708 | 0.0001 |  |
| Sex | -0.307±0.232 | 0.374 | 0.1866 |  |
| Age*Sex | 0.003±0.005 | 0.120 | 0.4543 |  |
| **FG** | **-0.057±0.020** | **1.713** | **0.005** | **0.1418** |
|  |  |  |  |  |
| **Model 3** |  |  |  |  |
| *Intercept* | *10.574±0.193* | *639.837* | *0.0001* |  |
| Age | - 0.012±0.003 | 5.207 | 0.0001 |  |
| Sex | - 0.301±0.233 | 0.354 | 0.1987 |  |
| Age*Sex | 0.003±0.667 | 0.067 | 0.5759 |  |
| **HbA_1c_** | **-0.020 ±0.031** | **0.092** | **0.5114** | **0.1211** |
|  |  |  |  |  |
| **Model 4** |  |  |  |  |
| *Intercept* | *10.420±0.220* | *491.932* | *0.0001* |  |
| Age | - 0.013±0.003 | 5.632 | 0.0001 |  |
| Sex | - 0.306±0.238 | 0.363 | 0.1995 |  |
| Age*Sex | 0.002±0.004 | 0.060 | 0.6015 |  |
| **SBP** | **0.001 ±0.002** | **0.046** | **0.6456** | **0.1189** |

Abbreviations: FG, fasting glucose; HbA_1c,_ glycosylated hemoglobin; HOMA-IR, homeostasis model assessment of insulin resistance; LTL, leukocytes telomeres length; SBP, systolic blood pressure; Type III SS: type III sum of squares
