# Supplementary material for: Association of Insulin Resistance, Arterial Stiffness and Telomere Length in Adults Free of Cardiovascular Diseases
Source: PLoS One. 2015 Aug 26;10(8):e0136676. doi: 10.1371/journal.pone.0136676 (PMC4550423; doi:10.1371/journal.pone.0136676)
Supplement: S2 Fig — (DOCX) [file pone.0136676.s004.docx]

S2 Fig. Histogram plot of leukocyte telomere length (LTL) values distribution
