# Supplementary material for: Association of Insulin Resistance, Arterial Stiffness and Telomere Length in Adults Free of Cardiovascular Diseases
Source: PLoS One. 2015 Aug 26;10(8):e0136676. doi: 10.1371/journal.pone.0136676 (PMC4550423; doi:10.1371/journal.pone.0136676)
Supplement: S4 Fig — (DOCX) [file pone.0136676.s006.docx]

S4 Fig. Scatter plots of leukocyte telomere length (LTL) as a function of Homeostasis model assessment of insulin resistance (HOMA-IR),y = 9.957-0.067HOMA-IR, R^2^ = 0.0962
